# Evaluation of the prevalence and severity of temporomandibular disorders and oral behaviors in a group of Iranian bodybuilders: a cross-sectional study

**DOI:** 10.1186/s12903-025-07067-y

**Published:** 2025-11-04

**Authors:** Hamed Mortazavi, Homa Mirzaei, Nazli Rastkar, Farid Zayeri, Aliparsa Rastkar

**Affiliations:** 1 Department of Oral Medicine, School of Dentistry, Shahid Beheshti University of Medical Sciences, Tehran, Iran; 2 Dental Research Center, Research Institute for Dental Sciences, School of Dentistry, Shahid Beheshti University of Medical Sciences, Tehran, Iran; 3 Proteomics Research Center, Department of Biostatistics, Faculty of Allied Medical Sciences, Shahid Beheshti University of Medical Sciences, Tehran, Iran

**Keywords:** Temporomandibular disorders, Iran, Resistance training, Weight lifting

## Abstract

**Background:**

Temporomandibular disorders (TMDs) are the most common non-dental orofacial pain conditions. Due to the potential elevated risk of TMD in bodybuilders and the lack of research in the Iranian population, this study aimed to investigate the prevalence and severity of TMDs and oral behaviors and compare associated variables among Iranian bodybuilders for the first time.

**Methods:**

This cross-sectional study was conducted on 378 bodybuilders aged 18–60 years old who had been actively training for at least 6 months. The prevalence and severity of TMD were assessed using the Fonseca questionnaire, while oral parafunctional behaviors were evaluated using an Oral Behaviors Checklist (OBC). Ultimately, the study results were analyzed using the Chi-squared test, Fisher’s exact test, and Pearson correlation.

**Results:**

The results of the Fonseca questionnaire showed that 48.2% of the participants have TMD. No significant relationship was found between the prevalence and severity of TMD and age, gender, months of bodybuilding, number of training hours per week, the heaviest weight used in the bench press, and Body Mass Index (BMI). According to the results of the OBC, 100.0% of the participants had oral parafunctional behaviors, and a significant relationship was observed between oral parafunctional behaviors and gender (*P* = 0.047) and the heaviest weight used in the bench press exercise (*P* = 0.002). According to the study, a significant correlation was observed between the prevalence of TMD and the prevalence of oral parafunctional behaviors (*P* = 0.02, *r* = 0.245).

**Conclusion:**

About half of the bodybuilders have TMD, and all of them have oral parafunctional behaviors.

## Introduction

In recent years, temporomandibular disorders (TMDs) have been recognized as a global health issue. TMD is the most common orofacial pain of non-dental origin, and the challenges of early diagnosis and inconsistent treatment plans are considered major issues of this condition [1]. These disorders are characterized by pain in the preauricular area, temporomandibular joint (TMJ), and masticatory muscles, limited movement, deviation of the lower jaw, and TMJ sounds during its function [2].

Oral parafunctional behaviors as a subgroup of oral behaviors, such as clenching (grinding teeth) among resistance training athletes, have increased to improve performance during workouts [3]. This oral parafunctional behavior is a risk factor for the progression of TMD; as the frequency of this action increases, it is associated with a higher occurrence of musculoskeletal pain. Therefore, the risk of developing TMDs in athletes is higher than in non-athletes [3].

One of the important stages in research is the collection of data using precise measurement tools [4]. One of these tools is the use of questionnaires whose validity and reliability have been confirmed. The Fonseca Anamnestic Index (FAI) questionnaire is widely used to assess the prevalence and severity of TMDs in clinical and community samples [5]. This questionnaire is a low-cost and simple tool for use in epidemiological studies and screening for TMD in non-patient populations. The original language of the questionnaire is Portuguese, and it has been translated and validated into other languages, including Persian, English, Spanish, Arabic, and others [4, 6]. The Oral Behaviors Checklist (OBC) is a scale for identifying and measuring the frequency of excessive jaw use, which is now recommended for assessing oral behaviors. Additionally, the OBC is considered a tool for assessing the main risk factor for TMD [7].

The prevalence of TMD is approximately 56.5% in bodybuilders [8]. Due to the high importance of early identification of patients with TMD among bodybuilders and other resistance-trained athletes and the lack of research on this topic in the Iranian population, this investigation would be the first report on the prevalence of TMDs and oral behaviors in the Iranian bodybuilding population. The aim of this study is to investigate the prevalence and severity of TMDs and oral behaviors and to compare the related factors in these individuals.

## Materials and methods

### Data collection

#### Study population

The present cross-sectional study collected data from athletes engaged in bodybuilding and resistance training exercises at gyms located in Tehran. Participants were included or excluded based on the following criteria:

Inclusion Criteria:

1. Participants were between 18 and 60 years old. These ages were chosen because they are the ones with the largest number of healthy volunteers who practice resistance training [3].
2. They had been continuously engaged in bodybuilding or resistance training for at least 6 months and exercised at least 2 days per week for at least 1 h each session [8].
3. They had obtained at least a high school diploma. Therefore, they have a better understanding of the questions related to the Fonseca questionnaire and the OBC.
4. The informed consent form should have been filled out independently.

Exclusion Criteria:

1. Individuals diagnosed with cognitive or psychiatric conditions that impaired their ability to understand and complete the questionnaire, such as schizophrenia or cognitive disorders [8, 9]. Since the responses of these groups of participants to the Fonseca questionnaire and the OBC are not reliable.
2. Those with a history of surgery or severe trauma to the TMJ, including procedures like condylectomy [10].
3. Completely edentulous patients or those using removable dentures. Since occlusal disorders or edentulism may cause symptoms that mimic TMD [11, 12].
4. Individuals diagnosed with joint diseases, such as rheumatoid arthritis or osteoarthritis [13]. Considering that these disorders may affect the TMJ.
5. Participants suffering from painful conditions, including fibromyalgia, chronic headaches, neck pain, back pain, pericoronitis, or otitis [3].
6. Individuals currently undergoing treatment for psychological disorders or taking psychiatric medications [14].
7. Those who are currently experiencing general health issues, especially those with general neurological issues (sensory or reflex changes, weakness, etc.) or suffering from uncontrolled hormonal disease (diabetes, thyroid, or parathyroid disease, etc.) [15].

#### Collection methods

The Fonseca questionnaire is a useful tool to evaluate the TMD severity in patients, and the Persian translation of this questionnaire also has validity and reliability [16–18]. In order to examine the prevalence and severity of oral parafunctional behaviors, the OBC was used. This checklist is a scale for identifying and measuring the frequency of excessive jaw use [19, 20]. In addition, its validity and reliability have been confirmed in Persian-speaking populations by Hemmati et al. [21].

The data was collected using the Fonseca questionnaire, OBC, and demographic information, including sex, age, months of training in bodybuilding, hours of training per week, the heaviest weight used in the bench press exercise, and Body Mass Index (BMI), through online and printed forms within the range of 21 st August 2023 to 31 st May 2024. The questionnaires were shared either in person or online in the social media groups and channels of gyms across Tehran. In this study, an application was used that allows each IP address to fill out the online questionnaire only once to ensure data accuracy. In recent years, the use of online questionnaires for data collection has gained significant popularity because it allows for the collection of a large amount of information in a shorter time and with less energy and cost. In this regard, Meyerson and Tryon, and Lewis et al. have shown that internet-based surveys provide data that is at least as trustworthy, valid, and of similar quality as data acquired via paper-and-pencil surveys [22, 23]. All respondents meeting the study’s inclusion requirements were included using the convenience sampling method. At the beginning of the questionnaire, participants were asked whether they had a diagnosed psychological problem or systemic conditions associated with joint disorders, if they had any history of surgery or trauma related to the TMJ, whether they were completely edentulous or had removable dentures, and if they had painful disorders such as fibromyalgia, headaches, neck pain, back pain, pericoronitis, and otitis. Furthermore, the form included a checkbox to notify participants about the research and ask them for consent; all records with the unchecked consent box were excluded from the study.

The Fonseca questionnaire consists of ten questions related to maximum mouth opening restriction, lateral movements of the mandible, pain in masticatory muscles, referral pain in the head, neck, and shoulder, feeling pain in the TMJ and around the ears, noises in the TMJ during movements, behaviors related to unbalanced occlusion, and anxiety. Each question had three options: ‘Yes,’ ‘Sometimes,’ and ‘No,’ where, by choosing each one, the patient was given 10, 5, and 0 points, respectively. The severity score for a patient was determined by summing points of all questions and was classified as Normal (0–15 points), Mild (20–40 points), Moderate (45–65 points), and Severe (70–100 points). Any patient with a severity score higher than the normal grade was considered a patient with TMD.

The OBC includes 21 questions that are divided into two categories: (1) Activities during sleep (including 2 questions) asking about clenching or grinding teeth when asleep and sleeping in a position that puts pressure on the jaw, (2) Activities during waking hours (including 19 questions) asking about clenching or grinding teeth during waking hours; pressing, touching, or holding teeth together other than while eating; tense muscles without clenching or bringing teeth together; holding jaw forward to the side; pressing tongue forcibly against teeth; placing tongue between teeth; holding jaw in a rigid or tense position; holding between the teeth or biting objects; chewing gum; playing a musical instrument that involves use of mouth or jaw; leaning with your hand on the jaw; chewing food only on one side; eating between meals; sustained talking; singing; and yawning. Each question had five options: ‘None of the time,’ ‘A little of the time,’ ‘Some of the time,’ ‘Most of the time,’ and ‘All of the time,’ where, by choosing each one, the patient was given 0, 1, 2, 3, and 4 points, respectively. The severity score for a patient was determined by summing points of all questions and was classified as without oral parafunctional behaviors (0 points), mild oral parafunctional behaviors (1–24 points), and severe oral parafunctional behaviors (25–84 points). Any patient with a severity score higher than zero was considered a patient with oral parafunctional behaviors.

### Sample size

Based on similar studies, the prevalence of TMDs in the population of athletes engaged in professional and recreational training was on average close to 57.6% [8]. For this reason, to estimate this prevalence in the population with 80% confidence and a margin of error of 0.05, a sample size of 375 people was considered. The community of fitness clubs was divided into two categories, specifically for women and men. In each category, athletes were randomly assigned to the study to ensure that the samples were homogeneous in terms of gender distribution.

### Statistical tests

This study used IBM SPSS 23.0 for statistical analysis. Chi-square and Fisher’s exact tests were used for data analysis. To examine the relationship between the severity of TMD and the severity of oral parafunctional behaviors, Pearson correlation was used. P-values lower than 0.05 were considered significant.

## Results

###  Description of population

Of the 384 completed questionnaires, two questionnaires were filled out incompletely, three subjects trained under two hours per week, and one subject was under 18 years old. From the others, the 378 remaining subjects qualified for inclusion in the study. The demographic information of the participants is summarized in Table 1. The mean age of the study population was 27.32 ± 7.05 years old. In the gender distribution of the participants present in the study, the male-to-female ratio in the community was close to one; out of the 378 participants present, 192 were men (50.8%) and 186 were women (49.2%) (Table 1).

**Table 1:** Demographic information of the study participants

Variable	Values			
	Minimum	Maximum	Average	Standard deviation
Age (years)	18.00	55.00	27.32	7.05
Weight (kilograms)	41.00	106.00	71.42	13.45
Height (centimeters)	148.00	200.00	172.54	9.22
Body Mass Index (kg/m2)	15.62	33.06	23.83	3.16
Duration of bodybuilding (months)	6.00	264.00	49.41	44.68
Duration of training per week (hours)	2.00	20.00	5.33	2.28
The heaviest weight used in bench press (kilograms)	10.00	200.00	45.74	31.99
Sex	Frequency (percentage)			
Men	192 (50.8%)			
Women	186 (49.2%)			

### Tests results

#### Prevalence and severity of TMD and oral parafunctional behaviors

According to the responses given by the participants to the Fonseca questionnaire, 42.8% of the participants had TMD, with 34.9% in the mild TMD group and 7.9% in the moderate TMD group.

Based on the responses of the participants to the OBC, all individuals had at least one oral parafunctional behavior, with 73.5% in the mild oral parafunctional behaviors group and 26.5% in the severe oral parafunctional behaviors group (Table 2).

**Table 2:** The prevalence and severity of TMD and oral parafunctional behaviors among the participants in the study based on the scores obtained in the Fonseca questionnaire and OBC

Type of disorder	Severity	Frequency (percentage)
TMD	Mild	132 (34.9)
	Moderate	30 (7.9)
	Severe	(−)
Total number of individuals with TMD	-	162 (42.8)
Oral parafunctional behaviors	Mild parafunctional behaviors	278 (73.5)
	Severe parafunctional behaviors	100 (26.5)
Total number of individuals with oral parafunctional behaviors	-	378 (100.0)

#### Prevalence and severity of TMD and oral parafunctional behaviors based on participants’ age groups

No significant relationship was observed between the prevalence and severity of TMD (*P* = 1.000) and oral parafunctional behaviors (*P* = 0.439) and different age groups (Table 3).

**Table 3:** Prevalence and severity of TMD and oral behaviors in athletes with TMD and/or oral parafunctional behaviors based on different age groups

Age groups	Frequency and severity of TMD				Frequency and severity of oral behaviors		
	Mild	Moderate	Severe	Total	Mild	Severe	Total
	Frequency (percentage)	Frequency (percentage)	Frequency (percentage)	Frequency (percentage)	Frequency (percentage)	Frequency (percentage)	Frequency (percentage)
Less than 30 years	94 (58.0)	22 (13.6)	-	116 (71.6)	195 (51.6)	75 (19.8)	270 (71.4)
More than 30 years	38 (23.5)	8 (4.9)	-	46 (28.4)	83 (22.0)	25 (6.6)	108 (28.6)
Total	132 (81.5)	30 (18.5)	-	162 (100.0)	278 (73.6)	100 (26.4)	378 (100.0)
P-value^*^	1.000				0.439		

^*^Chi-squared test

#### Prevalence and severity of TMD and oral parafunctional behaviors based on gender distribution of the participants

No significant relationship was observed between the prevalence and severity of TMD and the gender distribution of the participants (*P* = 0.317) (Table 4). A significant relationship was observed between the severity of oral parafunctional behaviors and the gender distribution of the participants (*P* = 0.047) (Table 4).

**Table 4:** Prevalence and severity of TMD and oral behaviors in participants with TMD and/or oral parafunctional behaviors based on gender distribution

Sex	Frequency and severity of TMD				Frequency and severity of oral behaviors		
	Mild	Moderate	Severe	Total	Mild	Severe	Total
	Frequency (percentage)	Frequency (percentage)	Frequency (percentage)	Frequency (percentage)	Frequency (percentage)	Frequency (percentage)	Frequency (percentage)
Male	65 (40.1)	18 (11.1)	-	83 (51.2)	150 (39.7)	42 (11.1)	192 (50.8)
Female	67 (41.4)	12 (7.4)	-	79 (48.8)	128 (33.9)	58 (15.3)	186 (49.2)
Total	132 (81.5)	30 (18.5)	-	162 (100.0)	278 (73.6)	100 (26.4)	378 (100.0)
P-value^*^	0.317				0.047		

^*^Chi-squared test

#### Prevalence and severity of TMD and oral parafunctional behaviors based on participants’ months of bodybuilding

No significant relationship was observed between the prevalence and severity of TMD (*P* = 0.838) and oral parafunctional behaviors (*P* = 0.236) and participants’ months of bodybuilding (Table 5).

**Table 5:** The prevalence and severity of TMD and oral behaviors in athletes with TMD and/or oral parafunctional behaviors based on the duration of bodybuilding (months)

Duration of bodybuilding (month)	Frequency and severity of TMD				Frequency and severity of oral behaviors		
	Mild	Moderate	Severe	Total	Mild	Severe	Total
	Frequency (percentage)	Frequency (percentage)	Frequency (percentage)	Frequency (percentage)	Frequency (percentage)	Frequency (percentage)	Frequency (percentage)
More than 48	79 (48.8)	17 (10.5)	-	96 (53.9)	161 (42.6)	65 (17.2)	226 (59.8)
Less than 48	53 (32.7)	13 (8.0)	-	66 (40.7)	117 (31.0)	35 (9.2)	152 (40.2)
Total	132 (81.5)	30 (18.5)	-	162 (100.0)	278 (73.6)	100 (26.4)	378 (100.0)
P-value^*^	0.838				0.236		

^*^Chi-squared test

#### Prevalence and severity of TMD and oral parafunctional behaviors based on participants’ number of training hours per week

No significant relationship was observed between the prevalence and severity of TMD (*P* = 0.229) and oral parafunctional behaviors (*P* = 0.815) and participants’ number of training hours per week (Table 6).

**Table 6:** Prevalence and severity of TMD and oral behaviors in athletes with TMD and/or oral parafunctional behaviors based on weekly training duration (hours)

Weekly training duration (hours)	Frequency and severity of TMD				Frequency and severity of oral behaviors		
	Mild	Moderate	Severe	Total	Mild	Severe	Total
	Frequency (percentage)	Frequency (percentage)	Frequency (percentage)	Frequency (percentage)	Frequency (percentage)	Frequency (percentage)	Frequency (percentage)
More than 5	58 (35.8)	17 (10.5)	-	75 (46.3)	121 (32.0)	45 (11.9)	166 (43.9)
Less than 5	74 (45.7)	13 (8.0)	-	87 (53.7)	157 (41.5)	55 (14.6)	212 (56.1)
Total	132 (81.5)	30 (18.5)	-	162 (100.0)	278 (73.5)	100 (26.5)	378 (100.0)
P-value^*^	0.229				0.815		

^*^Chi-squared test

#### Prevalence and severity of TMD and oral parafunctional behaviors based on participants’ heaviest weight used in the bench press exercise

No significant relationship was observed between the prevalence and severity of TMD and participants’ heaviest weight used in the bench press (*P* = 0.103) (Table 7). A significant relationship was observed between the severity of oral parafunctional behaviors and participants’ heaviest weight used in the bench press exercise (*P* = 0.002) (Table 7).

**Table 7:** The prevalence and severity of TMD and oral behaviors in athletes with TMD and/or oral parafunctional behaviors based on the heaviest weight used in the bench press (kilograms)

The heaviest weight used in the bench press (kilograms)	Frequency and severity of TMD				Frequency and severity of oral behaviors		
	Mild	Moderate	Severe	Total	Mild	Severe	Total
	Frequency (percentage)	Frequency (percentage)	Frequency (percentage)	Frequency (percentage)	Frequency (percentage)	Frequency (percentage)	Frequency (percentage)
More than 45	80 (49.4)	13 (8.0)	-	93 (57.4)	140 (37.0)	68 (18.0)	208 (55.0)
Less than 45	52 (32.1)	17 (10.5)	-	69 (42.6)	138 (36.5)	32 (8.5)	170 (45.0)
Total	132 (81.5)	30 (18.5)	-	162 (100.0)	278 (73.5)	100 (26.5)	378 (100.0)
P-value^*^	0.103				0.002		

^*^Chi-squared test

#### Prevalence and severity of TMD and oral parafunctional behaviors based on participants’ BMI

No significant relationship was observed between the prevalence and severity of TMD (*P* = 0.308) and oral parafunctional behaviors (*P* = 0.901) and participants’ BMI (Table 8).

**Table 8:** The prevalence and severity of TMD and oral behaviors in athletes with TMD and/or oral parafunctional behaviors based on BMI

BMI	Frequency and severity of TMD				Frequency and severity of oral behaviors		
	Mild	Moderate	Severe	Total	Mild	Severe	Total
	Frequency (percentage)	Frequency (percentage)	Frequency (percentage)	Frequency (percentage)	Frequency (percentage)	Frequency (percentage)	Frequency (percentage)
Less than 25 (normal and underweight)	71 (43.8)	20 (12.3)	-	91 (56.1)	173 (45.8)	64 (16.9)	237 (63.7)
25 to 30 (overweight)	56 (34.6)	10 (6.2)	-	66 (40.8)	98 (25.9)	33 (8.7)	131 (34.6)
More than 30 (obese)	5 (3.1)	0 (0.0)	-	5 (3.1)	7 (1.9)	3 (0.8)	10 (2.7)
Total	132 (81.5)	30 (18.5)	-	162 (100.0)	278 (73.6)	100 (26.4)	378 (100.0)
*P*-value^*^	0.308				0.901		

#### Distribution of TMD severity among the participants in the study, categorized by the classification of oral parafunctional behaviors

We observed a significant relationship between the severity of TMD and oral parafunctional behaviors (*P* = 0.02). The correlation analysis of TMD and OBC with a Pearson correlation coefficient of 0.245 showed that there is a weak to low-moderate correlation between TMD and OBC, indicating a direct relationship between the severity of TMD and oral parafunctional behaviors (Table 9).

**Table 9:** Distribution of TMD severity among participants in the study categorized by oral parafunctional behaviors

**Severity of oral behaviors based on OBC**	**Severity of TMD**				**P-value**	**Pearson correlation coefficient**
	**Mild**	**Moderate**	**Severe**	**Total**		
	Frequency (percentage)	Frequency (percentage)	Frequency (percentage)	Frequency (percentage)	0.002	0.245
Mild	81 (50.0)	9 (5.5)	-	90 (55.5)		
Severe	51 (31.5)	21 (13.0)	-	72 (44.5)		
Total	132 (81.5)	30 (18.5)	-	162 (100.0)		

^*^P values are calculated based on the Chi-squared test

## Discussion

### Main finding

Between 2011 and 2023, according to the available literature, the prevalence of TMD in the Iranian population was evaluated as 28.3–43.6% [24–27]. The prevalence of TMD among bodybuilders with a mean age of 27.32 ± 7.05 years old in the present study was 42.8%. This distribution indicates a relatively high prevalence of TMD among bodybuilders compared to the general population, which may be due to intense physical activities and oral parafunctional behaviors associated with these activities. No significant relationship was found between the prevalence and severity of TMD and age, gender, months of bodybuilding training, number of training hours per week, the heaviest weight used in the bench press, and BMI. We also discovered that based on the OBC, all participants had at least one oral parafunctional behavior (73.5% mild, 26.5% severe), which was not evaluated in any previous studies in this population. A significant relationship was observed between oral parafunctional behaviors and gender (*P* = 0.047) and the heaviest weight used in the bench press exercise (*P* = 0.002). According to the study, a significant correlation was observed between the prevalence of TMD and the prevalence of oral parafunctional behaviors (*P* = 0.02, *r* = 0.245).

### Related studies and other findings

In the present study, age groups, gender, months of bodybuilding, number of training hours per week, heaviest weight used in the bench press exercise, and BMI were evaluated among individuals with TMD and oral parafunctional behaviors.

No significant relationship was found between the age groups and the prevalence and severity of TMD. This finding is consistent with the results of the study by Mansour Ibrahim Alrowdan et al., which did not find a significant relationship between the age of Saudi weightlifters and the prevalence and severity of TMD [28].

Male patients with TMD outnumbered female patients, but the difference was not significant. Although in most studies related to TMD, the number of women reported is higher compared to men, there are also contradictory reports that either found no difference between the two genders or conversely reported a higher prevalence in men [29–31]. This finding is not consistent with the study by Mansour Ibrahim Alrowdan et al. He showed that the prevalence of TMD based on the Fonseca questionnaire in female weightlifters was significantly higher than in male weightlifters in all mild, moderate, and severe TMD groups [28]. This difference could be due to the differences between the Iranian and Saudi Arabia societies. Regarding the difference in results between this study and the study in Saudi Arabia, it should be noted that TMD is a multifactorial disorder. In addition to local causes such as various microtraumas, bruxism, etc., other factors like genetic and racial differences, anatomical factors such as joint morphology and muscle attachments, and even individuals’ psychological conditions can play a role in its occurrence or severity. This might be associated with different results in various communities [32].

No significant relationship was observed between the months of bodybuilding and the prevalence and severity of TMD. This conclusion aligns with the findings of the research conducted by Kaminiecki et al., which did not observe a significant relationship between the years of training in CrossFit athletes and the prevalence of TMD [33]. This result might be explained by the fact that, generally, those who train for longer periods are more professional. This professionalism is not limited to the physical aspect; these individuals are likely to have more information and awareness regarding the potential side effects of the sport that they practice. This can play a role in the prevention of the cumulative effects of variables related to the development of TMD by controlling parafunctional habits or performing timely self-treatments. Meanwhile, more practice enables these individuals to be more precise in using tools and weights and to avoid unnecessary or inappropriate pressure, which in turn plays an important role in reducing oral parafunctional behaviors during exercise. Similar findings, especially regarding the role of coaches in controlling the performance of professional athletes, have been presented by Freiwald et al. It has been suggested that this leads to a lower prevalence of maxillofacial problems in these athletes [8]. Of course, determining these relationships requires further studies.

No significant relationship was found between the number of training hours per week and the prevalence and severity of TMD. This finding somewhat contradicts the results of the study by Friedman Rubin et al.; based on their results, the prevalence of TMD symptoms in athletes engaged in intense resistance training (those who trained at least 4 sessions per week) was higher than in recreational athletes (those who trained less than 4 sessions per week) [15]. The reason for this difference could be due to variations in the studied populations; in our study, both male and female groups were examined, whereas Friedman Rubin et al. only studied female athletes.

No significant association was detected between the heaviest weight used in the bench press exercise and the prevalence and severity of TMD. As no study has investigated the relationship between the weight of dumbbells used in bodybuilding and the frequency and severity of TMD, this result remains incomparable.

There was no significant relationship between BMI and the prevalence and severity of TMD. While Miettinen et al. showed that regular physical activity and fitness can act as protective factors against TMD pain. The results of this study showed that individuals who exercise less than once a week or are overweight are more likely to experience TMD symptoms [34]. The reason for this difference could be due to variations in the studied populations, the number of individuals examined, and the criteria used to determine TMD symptoms. Miettinen et al. conducted their study on 8,685 Finnish soldiers, regardless of whether they were athletes or non-athletes, and used a 6-question questionnaire to assess TMD symptoms [34]. But our study was conducted on 378 Iranian bodybuilders using the Fonseca questionnaire.

Based on the results of the OBC, all participants had oral parafunctional behaviors. We were unable to find a similar study examining the prevalence and severity of oral parafunctional behaviors based on the OBC in the Iranian general and athlete population, thereby preventing a comparison of this result.

No significant relationship was observed between the age groups and the prevalence and severity of oral parafunctional behaviors. This finding is similar to the result of Melchior’s study, which showed that the prevalence of oral parafunctional behaviors had a negative correlation with age in TMD patients [35]. There is also evidence that among oral parafunctional behaviors, frequency of bruxism decreases with age [36]. On the other hand, Michelotti showed that those oral parafunctional behaviors that predispose the disc to displacement decrease with age [37]. According to the study by Lee et al., it has been determined that those in younger age groups have more psychological issues and physical tensions, which may increase the occurrence of oral parafunctional behaviors [38].

No significant relationship was observed between the months of bodybuilding and the training hours per week with the prevalence and severity of oral parafunctional behaviors. We could not locate a comparable study that investigated the prevalence and severity of oral parafunctional behaviors concerning these factors, which would not allow for a comparison.

We observed a significant relationship between gender distribution and the prevalence and severity of oral parafunctional behaviors, suggesting a greater vulnerability of women to oral parafunctional behaviors. We were unable to find a similar study examining the prevalence and severity of oral parafunctional behaviors based on the gender of athletes, making a comparison impractical.

A significant relationship was found between the heaviest weight used in the bench press exercise and the prevalence and severity of oral parafunctional behaviors. This finding is similar to the results of the study by Júnior et al., which showed that the effectiveness of the chewing system decreases after resistance training [10]. This decrease in chewing effectiveness may be due to the fatigue of the masticatory muscles and the onset of anaerobic metabolism after exercise. It can be said that with the increase in weight, the individual may exert more pressure on their masticatory muscles during the exercise, associating with fatigue and a decrease in the capacity of these muscles after the workout, ultimately resulting in insufficient strength to perform certain oral parafunctional behaviors. On the other hand, it should be noted that the number of individuals who trained with weights over 100 kg was much lower than the number of individuals who trained with weights under 50 kg, and this difference in frequency could be another reason for this finding. Additionally, a study by Freiwald et al. showed that the prevalence of TMD among competitive female athletes is lower than that among amateur athletes and non-athletic women [8]. This difference may be due to better supervision and appropriate physiotherapy among professional athletes, which can play a protective role against the occurrence of TMD. It can be said that individuals who use heavier weights to improve their physical and muscular strength require supervision from experienced trainers. These coaches recommend protective methods, such as using mouthguards or physiotherapy, to this group of individuals to prevent potential injuries, which helps in preventing the formation or exacerbation of oral parafunctional behaviors.

We found no significant relationship between BMI and the prevalence and severity of oral parafunctional behaviors. While the study by Miettinen et al. showed that regular physical activity and fitness can act as protective factors against TMD pain, individuals who exercise less than once a week or are overweight are more likely to experience TMD symptoms [34]. The reason for this difference could be attributed to the differences within the studied communities. While Miettinen studied 8,685 Finnish soldiers without taking into account their athletic status, our study focused on Iranian bodybuilders.

Our study’s results revealed a significant relationship between the severity of TMD and the severity of oral parafunctional behaviors. It shows that mild TMD and oral parafunctional behaviors are directly related. This finding is consistent with the study by Karabicak and Kanik, which showed that the severity of TMD correlates with oral parafunctional behaviors, neck pain, and neck function [39].

Pearson correlation analysis between TMD and OBC showed a weak to low-moderate correlation between these two variables. This finding clearly indicates that oral parafunctional behaviors may be associated with the progression and severity of TMD in bodybuilders.

In-person examinations of individuals can yield more accurate results and reduce self-reported data bias, but considering the relatively large number of participants and their geographical dispersion, the indirect methods were used. Also, considering these limitations, we used convenience sampling as our sampling strategy.

Based on the results of our study and similar studies, it seems that proper supervision and management of bodybuilders may contribute to reducing the prevalence and severity of TMD and oral parafunctional behaviors among them [8]. It is possible to reduce the incidence of these complications by educating athletes, coaches, and physiotherapists. Additionally, by effective communication between athletes and dentists, early diagnosis of such disorders can be achieved. This is because the prognosis of TMD treatment is time-dependent, and the sooner it is diagnosed, the sooner it can be treated [40].

This study has findings that have been referenced in similar studies. Therefore, in these cases, the author has referred to potential cause-and-effect relationships that should be examined further in future research. Furthermore, we recommend further research about the relationship between resistance training and temporomandibular joint disorders in various populations, as well as their various impacts on oral-facial health. Moreover, considering that factors such as the level of stress and anxiety in individuals can affect parafunctional habits and the severity and prevalence of TMD, the impact of these variables should also be examined in this group of individuals.

Considering that there are no studies of TMD in Iranian bodybuilders, studies could be conducted in the future using more precise research tools, particularly the use of clinical indices through in-person examinations or the DC/TMD (Diagnostic Criteria for Temporomandibular Disorders) diagnostic system. TMD among athletes training in various subgroups of bodybuilding can be examined and compared. Bodybuilders with TMD and those without TMD can be compared by considering their training programs and oral behaviors, as well as by comparing amateur and professional bodybuilders. The role of professionals and coaching in the emergence of such problems can be investigated.

## Conclusion

The present study examined the prevalence and severity of temporomandibular joint disorders (TMD) and oral behaviors among Iranian bodybuilders for the first time. Our results showed that almost half of the bodybuilders have TMD, and all of them have at least one oral behavior.

## Data Availability

Further information on the data set and materials is available from the corresponding author upon reasonable request.

## Abbreviations

TMDs: Temporomandibular Disorders.
OBC: Oral Behaviors Checklist.
BMI: Body Mass Index.
TMJ: Temporomandibular Joint.
FAI: Fonseca Anamnestic Index.
